# Refining patient selection for next-generation immunotherapeutic early-phase clinical trials with a novel and externally validated prognostic nomogram

**DOI:** 10.3389/fimmu.2024.1323151

**Published:** 2024-01-15

**Authors:** Agnese Losurdo, Angelo Dipasquale, Laura Giordano, Pasquale Persico, Elena Lorenzi, Antonio Di Muzio, Chiara Barigazzi, James Korolewicz, Aman Mehan, Oreoluwa Mohammed, Benhard Scheiner, David J. Pinato, Armando Santoro, Matteo Simonelli

**Affiliations:** ^1^ Department of Biomedical Sciences, Humanitas University, Milan, Italy; ^2^ Medical Oncology and Hematology Unit, IRCCS Humanitas Research Hospital, Humanitas Cancer Center, Milan, Italy; ^3^ Biostatistic Unit, IRCCS Humanitas Research Hospital, Humanitas Cancer Center, Milan, Italy; ^4^ Division of Cancer, Department of Surgery and Cancer, Imperial College London, Hammersmith Hospital, London, United Kingdom; ^5^ Division of Gastroenterology and Hepatology, Department of Medicine III, Medical University of Vienna, Vienna, Austria; ^6^ Division of Oncology, Department of Translational Medicine (DIMET), Università del Piemonte Orientale A. Avogadro, Novara, Italy

**Keywords:** immunotherapy, early-phases clinical trials, prognostic scores, next-generations immunotherapies, immune-related adverse events

## Abstract

**Introduction:**

Identifying which patient may benefit from immunotherapeutic early-phase clinical trials is an unmet need in drug development. Among several proposed prognostic scores, none has been validated in patients receiving immunomodulating agents (IMAs)-based combinations.

**Patients and methods:**

We retrospectively collected data of 208 patients enrolled in early-phase clinical trials investigating IMAs at our Institution, correlating clinical and blood-based variables with overall survival (OS). A retrospective cohort of 50 patients treated with IMAs at Imperial College (Hammersmith Hospital, London, UK) was used for validation.

**Results:**

A total of 173 subjects were selected for analyses. Most frequent cancers included non-small cell lung cancer (26%), hepatocellular carcinoma (21.5%) and glioblastoma (13%). Multivariate analysis (MVA) revealed 3 factors to be independently associated with OS: line of treatment (second and third vs subsequent, HR 0.61, 95% CI 0.40-0.93, p 0.02), serum albumin as continuous variable (HR 0.57, 95% CI 0.36–0.91, p 0.02) and number of metastatic sites (<3 vs ≥3, HR 0.68, 95% CI 0.48-0.98, p 0.04). After splitting albumin value at the median (3.84 g/dL), a score system was capable of stratifying patients in 3 groups with significantly different OS (p<0.0001). Relationship with OS reproduced in the external cohort (p=0.008). Then, from these factors we built a nomogram.

**Conclusions:**

Prior treatment, serum albumin and number of metastatic sites are readily available prognostic traits in patients with advanced malignancies participating into immunotherapy early-phase trials. Combination of these factors can optimize patient selection at study enrollment, maximizing therapeutic intent.

## 1 Introduction

Along with the advent of targeted agents and immunotherapy, designs and aims of oncology early-phase clinical trials (ep-CTs) have radically changed (1, 2). Historically, early-phase studies have represented the bridge between preclinical research and clinical development, assessing the safety profile of novel anticancer agents in small and unselected patient populations, with limited or absent therapeutic intent (1, 2). Recently, these straightforward objectives have evolved into a rather more ambitious set of purposes, such as exploring efficacy endpoints through expansion cohorts of specific tumors and molecularly-selected subgroups of patients (1, 2). With this paradigm shift, modern ep-CTs possess a well-recognized therapeutic intent, having also led to accelerated drug approval for oncological unmet needs (1–3). Nevertheless, selecting patients who are more likely to benefit from inclusion in ep-CTs remains a key issue to secure quality of life but integrity of clinical trial data as well.

In the past years, several scores have been built to predict benefit from inclusion into ep-CTs exploring safety of cytotoxic agents, either alone or combined to molecular targeted therapies (4–8). More recently, as immunotherapy has become the cornerstone of oncology drug development, a large variety of immunomodulating agents (IMAs) are being investigated across Phase I studies’ new wave, either as single agents or into combination-based regimens. Prognostic scoring systems specifically addressing outcome of patients treated into immunotherapeutic ep-CTs are strongly needed, helping clinicians to identify who might actually benefit from trial recruitment rather than being addressed to supportive care alone. The Gustave Roussy Immune Score (GRIm-Score) included three parameters (serum albumin, serum lactate dehydrogenase (LDH) and neutrophil-to-lymphocyte ratio (NLR)), identifying two prognostic groups of patients (9). Similarly, the MDACC group found other clinical factors predicting worse outcome in patients enrolled in early-phase studies with checkpoint inhibitors (10). However, the advent of next-generation immunotherapies, targeting novel immunological pathways over programmed death (ligand)-1 (PD-(L)1) and cytotoxic T-lymphocyte-associated protein 4 (CTLA-4), and the more and more frequent inclusion of historically immune-resistant tumors, are posing new challenges in terms of clinical utility of known prognostic scores.

In our study, we explored the prognostic impact of several clinical and blood variables in a large, retrospective, monocentric cohort of patients treated with IMAs in ep-CTs. Then, we developed and applied a new prognostic score based on three factors, confirming its validity in an external retrospective cohort. Finally, we made a nomogram to be used as a prognostic tool in daily clinical practice.

## 2 Methods

### 2.1 Patients

We retrospectively collected data of all consecutive patients with advanced solid tumors enrolled into immunotherapeutic ep-CTs at Humanitas Cancer Center from May 2014 to April 2021. All information was handled anonymously in a password-locked database. Patients enrolled from second line onwards and having received at least one cycle of experimental therapy were deemed suitable for analyses. We included different immunotherapeutic ep-CTs (first-in-man, dose escalation and Phase I a/b studies with expansion cohorts) exploring safety and antitumor activity of IMAs given either as monotherapy or in combination with other agents (immunotherapeutics, chemotherapies or biological agents). Clinical data were obtained retrospectively for each patient at entering in the trial, while dynamic variables, such as blood count, blood chemistry and steroid prescription with accurate dosage were collected also at six weeks (+/- 7 days), roughly after completing the first cycle of experimental therapy. The complete list of all variables analyzed in this study were reported in **Supplementary Table 1**. The local pathology service assessed programmed-death ligand 1 (PD-L1) expression on the most recent tumor sample before enrollment for those patients who still had archival tissue to be retrieved for analysis (n=111/173, 64%). PD-L1 expression was assessed through immunohistochemistry (IHC) with the Ventana kit (SP263 assay) and tumor cell (TC) expression defined as ≥50%, 1-49% and <1%. Response evaluation criteria in solid tumors (RECIST) and radiological assessment in neuro-oncology (RANO) criteria were used for response assessment in solid tumors and glioblastoma, respectively. Disease control rate (DCR) was defined as the sum of stable disease (SD), partial response (PR) and complete response (CR) as best response, meanwhile objective response rate (ORR) was defined as the sum of PR and CR. The validation cohort included patients treated into immunotherapeutic ep-CTs at the Developmental Cancer Therapeutics Unit of the Imperial College (Hammersmith Hospital, London, UK) from December 2019 to December 2022.

### 2.2 Statistical methods

Data were summarized as frequencies and proportions or as medians and ranges. Survival curves were generated using the Kaplan-Meier method. We assessed progression-free survival (PFS; time from inclusion in the clinical trial to progressive disease (PD) or death from any cause) and overall survival (OS) (time from inclusion in the clinical trial to death from any cause). Differences between groups were evaluated using the log-rank test. The Cox proportional hazards regression model was used to calculate the hazard ratios (HRs) and their 95% confidence intervals (CIs). After checking for correlation among variables, the final model was built considering all factors statistically significant at level p<0.2 (two sides) in the univariable setting and which confirmed their effect in the multivariable model at level p=0.05, (two sides). All these analyses were performed using SAS version 9.4 (SAS Institute Inc., Cary, NC, USA). The ultimate multivariable Cox regression model was used to construct the OS nomogram prognostic model via *rms* package in R version 3.4.4. The nomogram was formulated to predict the probability of 1- and 2-year overall survival (OS). Discriminatory power was analyzed by using Harrell’s C-index (HCI) to test the predictive ability for OS.

## 3 Results

### 3.1 Patients’ characteristics

A total of 208 patients affected by advanced solid tumors and enrolled in immunotherapeutic ep-CTs at our Phase I Unit were identified. All those cases treated in first-line setting were excluded, given their intrinsic better prognosis and the frequent association of IMAs to standard of care therapy. Thus, a total of 173 patients in second or further line of therapy were selected for analyses.

Patients’ characteristics are summarized in **Table 1**. The median age was 63 years (range 26-82) and the most common tumor types included non-small cell lung cancer (NSCLC) (n=45, 26%), hepatocellular carcinoma (HCC) (n=37, 21.5%), glioblastoma (GBM) (n=23, 13%), colo-rectal cancer (n=10, 6%) and breast cancer (n=9, 5%). Other rarer histologies included neuroendocrine tumor (NET) (2.9%), urothelial cancer (2.3%), prostate cancer (1.7%), ovarian cancer (1%), sarcoma (1%), head and neck squamous cell carcinoma (HNSCC) (1%), clear cell renal carcinoma (CCRC) (0.5%), SCC of the anal canal (0.5%) and endometrial cancer (0.5%). Most subjects underwent IMAs combined with another immunotherapeutic agent, TKI or chemotherapy (47.5%, 23% and 2.5%, respectively), while 47 patients (27%) received single-agent IMAs. A list of all targets of experimental agents is reported in **Supplementary Table 2**.

**Table 1:** Patients’ clinicopathological characteristics.

Variable	N (%)
Sex	
Male	98 (57)
Female	75 (43)
Age	
≤70	135 (78)
>70	38 (22)
ECOG PS	
0	104 (60)
1	69 (40)
Smoking habit	
No	65 (38.5)
Yes	104 (61.5)
Missing	4
Tumor type	
NSCLC	45 (26)
HCC	37 (21.5)
GBM	23 (13)
CRC	10 (6)
Breast cancer	9 (5)
Pleural mesothelioma	9 (5)
Melanoma	6 (3.5)
Pancreatic cancer	6 (3.5)
Gastro-esophageal cancer	6 (3.5)
Others	22 (13)
Therapy type	
IMA plus IMA	82 (47.5)
IMA single agent	47 (27)
IMA plus TKIs	40 (23)
IMA plus chemotherapy	4 (2.5)
Line of therapy	
2nd	95 (55)
3rd	47 (27)
≥4th	31 (18)
N metastatic sites	
1	46 (31)
2	51 (34)
3	36 (24)
≥4	17 (11)
Not applicable*	23
Best response	
SD	80 (46.5)
PR	14 (8)
CR	2 (1)
PD	77 (44.5)
Steroids pre-treatment	
>10 mg	14 (8)
≤10 mg	159 (92)
Albumin pre-treatment	
≥35 g/L	143 (84)
<35 g/L	27 (16)
LDH pre-treatment	
≤ULN	90 (71)
>ULN	37 (29)
NLR pre-treatment	
≤6	142 (82)
>6	31 (18)
PD-L1 status	
<1%	69 (62)
1-49%	32 (29)
≥50%	10 (9)
Missing	62

ECOG PS, Eastern Cooperative Oncology Group performance status; NSCLC, non-small cell lung cancer; HCC, hepatocellular carcinoma; GBM, glioblastoma; CRC, colorectal cancer; IMA, immunomodulating agent; TKIs, tyrosine kinase inhibitors; SD, stable disease; PR, partial response; CR, complete response; PD, progressive disease; LDH, lactate dehydrogenase; NLR, neutrophil-to-lymphocyte ratio.

*Refers to GBM patients.

### 3.2 Efficacy

The median follow-up was 46.6 months (range 1-90 months). Of the 173 patients analyzed, 165 experienced PD and 157 were dead at the time of analysis. Median PFS (mPFS) was 3.3 months (95% CI 2.6–3.9 months), with a median OS (mOS) of 9.8 months (95% CI 8.2-12.4 months) and survival rates at 6, 12, and 18 months of 69%, 45%, and 33%, respectively. Of the 169 subjects who discontinued study treatment, 132 (78.1%) were taken off protocol for progression, 20 (11.8%) for toxicity and 17 (10.1%) for other reasons, including consent withdrawal, clinical deterioration or physicians’ decision. In the overall population, 2 CRs and 15 PRs were observed, with a ORR of 9.8%. A total of 79 patients had SD as best response, leading to a DCR of 55.5%.

### 3.3 Adverse events

At least one AE was observed in 90% of patients (155/173), with the majority (112/173, 65%) considered as related to the experimental treatment (TRAEs). Among them, 83 patients experienced at least one grade 2 to 4 (G2-4) immune-related AE (ir-AEs), with 40 of them developing severe (G3-4) ir-AEs. The most common TRAEs were transaminase elevation (n=18), colitis and diarrhea (n=14), skin rash (n=12), hand-foot syndrome (n=11), hypertension (n=9) and lipase and amylase elevation (n=9). Most of the more frequent AEs, such as transaminase elevation, colitis/diarrhea, skin rash and lipase/amylase elevation, were deemed to be ir-AEs and treated accordingly. A list of the main TRAEs is available in **Supplementary Table 3**.

### 3.4 Univariate analysis

In the UVA, clinical factors associated with longer survival were early access to experimental treatment (second and third lines vs further lines of therapy; mOS 10.9 vs 5.9 months, p=0.007) and lower disease burden (≤2 metastatic sites vs >2) (mOS: 14.3 vs 6.2 months, p=0.01). Among blood-based parameters, a pre-treatment NLR>6 (mOS: 7.2 vs 10.8 months, p=0.04) and serum albumin both as continuous variable (for OS: HR 0.64, 0.41-0.99, p=0.04) and categorical value below 3.5 g/L (mOS: 5.9 vs 10.9 months, p=0.01) correlated with a negative outcome. A value of LDH above the upper value limit did not appear to be prognostic (p=0.7). Patients receiving more than 10 mg of prednisone or equivalent at trial inclusion showed significant decrease in OS compared to those taking less or no corticosteroids (mOS: 7.2 vs 10.9 months, p=0.05). No significant difference in survival was seen according to PD-L1 status among patients with high (≥50%), intermediate (1-49%) or negative (<1%) expression (p=1.0). The UVA results are summarized in **Table 2**.

**Table 2:** Univariate analysis results.

	N	Median PFS	P value	Median OS	P value
Sex					
Male	98	3.6	0.2	10.1	0.8
Female	75	2.8		9.8	
Age					
≤70	135	2.6	0.3	8.9	0.2
>70	38	5.1		14.6	
ECOG PS					
0	104	3.6	0.9	11.1	0.6
1	69	2.7		8.2	
Type of therapy					
IMA single agent	47	2.6	0.4	12.2	0.4
Combination therapy	126	3.9		9.5	
Line of therapy					
2^nd^-3^rd^	142	3.7	**0.001**	10.9	**0.007**
beyond	31	1.9		5.9	
N metastatic sites					
1-2	97	4	0.06	14.25	**0.01**
3+	53	2.4		6.2	
Steroids pre-treatment					
≤10 mg	159	3.6	**0.004**	10.8	**0.05**
>10 mg	14	1.6		7.2	
Albumin pre-treatment					
≥35 g/L	142	3.7	**0.04**	10.9	**0.01**
<35 g/L	27	2.1		5.9	
LDH pre-treatment					
≤ULN	90	3.8	0.9	10.9	0.7
>ULN	37	2.7		9.6	
NLR pre-treatment					
≤6	142	3.6	0.1	10.9	**0.04**
>6	31	1.9		7.3	
PD-L1 status					
<1%	69	2.6	0.4	8.1	1.0
1-49%	32	3.3		10.3	
≥50%	10	5.7		9.5	

In bold are statistically significant results.

ECOG PS, Eastern Cooperative Oncology Group performance status; IMA, immunomodulating agent; LDH, lactate dehydrogenase; NLR, neutrophil-to-lymphocyte ratio.

When not considering patients treated with anti-PD-(L)1 plus anti-CTLA-4 doublets, a type of combinational regimen already used in clinical practice and associated with improved survival, an exploratory analysis showed that those receiving a combination of IMAs only had a significant worse outcome compared to those treated with IMAs single-agent, IMAs plus TKIs and IMAs plus chemotherapy (mOS 7.2, 12.2, 16.9 and 24.6 months, respectively, p=0.003) (11–13) (**Supplementary Figure 1**). Focusing to the safety profile, the occurrence of any-grade TRAE and G2-4 ir-AEs was significantly associated to a better survival (mOS 11.1 vs 7.1 months, p=0.01; mOS 14.7 vs 7.9 months, p=0.01, respectively), with similar but not significant trend for severe (G3-4) ir-AEs (17.8 vs 9 months, p=0.2).

### 3.5 Multivariate analysis and development of a new prognostic score

In the MVA, three parameters remained independently correlated with better survival: pre-treatment serum albumin as a continuous variable, line of treatment and number of metastatic sites. The MVA results are summarized in **Table 3**.

**Table 3:** Multivariable analysis results.

Significant variables	HR for PFS	IC95	P value	HR for OS	IC95	P value
Number of metastatic sites (1-2 sites)	0.70	0.50 - 0.99	**0.05**	0.68	0.48 - 0.98	**0.04**
Albumin (g/dL) as continuous variable	0.55	035 - 0.88	**0.01**	0.57	0.36 - 0.91	**0.02**
Line of treatment (2^nd^/3^rd^ line)	0.50	0.32 - 0.74	**0.0008**	0.61	0.40 - 0.93	**0.02**
Corticosteroids dose >10 mg	1.82	0.79 - 4.19	0.16	1.51	0.66 - 3.45	0.32
Pre-treatment NLR>6	0.93	0.57 - 1.53	0.78	1.22	0.74 – 2.00	0.43

In bold are statistically significant variables.

**Figure 1:**
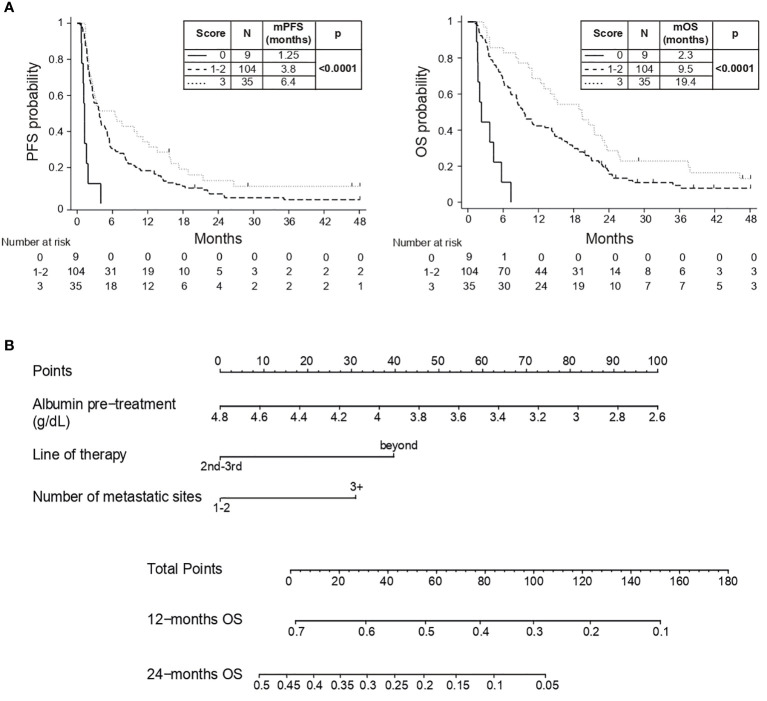
**(A)** PFS and OS Kaplan-Meyer curves; **(B)** Prognostic nomogram based on MVA. Example 1: a patient with serum albumin of 4.6 g/dL (10 points), in third-line of therapy (0 points) and with 1 metastatic site (0 points) will have a total score of 10 points with a 12-month OS of 68% and 24-month OS of 40%. Example 2: a patient with serum albumin of 3 g/dL (80 points), in fifth-line of therapy (40 points) and with 5 metastatic sites (30 points) will have a total score of 150 points with a 12-month OS of 10% and no 24-month OS.

Continuous serum albumin was categorized based on the median value (< vs ≥ 3.84 g/dL) and each favorable variable was assigned a numeric point. Patients were stratified in four different groups with increasing score and longer survival (p<0.0001). Subjects with a total score of 1 and 2 points showed similar outcome (mOS: 8.8 and 9.8 months) and were merged, obtaining three categories with distinct prognostic behavior (p<0.0001): patients with a score of 0 showed the worst outcome (mOS: 2.3 months), those with a score of 1-2 experienced better prognosis (mOS: 9.5 months) and those with a score of 3 had the longest survival (mOS: 19.4 months) (**Figure 1A**). Details on the variables distribution for each prognostic level are depicted in **Supplementary Table 4**.

### 3.6 Validation of our prognostic score in an external retrospective cohort

The external validation cohort included 50 patients treated into immunotherapeutic ep-CTs at the Developmental Cancer Therapeutics Unit of the Imperial College (Hammersmith Hospital, London, UK). Most prevalent cancer types included gynecological malignancies (40%), HNSCC (12%) and HCC (8%) and a total of 14 patients (28% of the cohort) received an IMAs-based combination. Main characteristics of the validation cohort are summarized in **Supplementary Table 5**. After a median follow-up of 6.4 months (range 0.7-19.8 months), the scoring system previously obtained confirmed its prognostic validity, with increasing score associated to better survival (mOS: 5.3 months vs 7.8 months vs not reached for 0, 1-2 and 3 points, respectively, p=0.008) (**Supplementary Figure 2**).

### 3.7 Prognostic nomogram based on MVA

Serum albumin, line of treatment and metastatic sites were combined in a handy nomogram, where increasing score was associated to expected shorten survival (HCI 0.63, 95% CI 0.57-0.69) (**Figure 1B** *).* Points (ranging from 0 to 100) are assigned to each continuous value of pre-treatment serum albumin (g/dL), line of therapy (second-third lines vs beyond) and number of metastatic sites (1-2 sites vs 3 or more); the obtained sum is graphically aligned to provide a specific 12-months and 24-months OS probability.

## 4 Discussion

In modern oncology, selecting patients for inclusion in ep-CTs remains challenging. In recent years, several prognostic scoring systems have been proposed to select candidates for ep-CTs, most of them were designed for patients treated with cytotoxic and targeted agents and only few for those receiving immunotherapy (4–10).

In our study, we assessed the prognostic relevance of a wide range of clinical and blood-based variables in a large cohort (n=208) of patients treated into immunotherapeutic ep-CT. We selected for the analysis subjects treated from second line on (n=173), given the fact that standard of care, usually included in first-line regimens, may hide the survival signal leaded by novel agents. Compared to previous reports, our cohort reflects modern Phase I trials landscape and appears unique for several reasons (9, 10, 14). Firstly, a heterogeneous spectrum of tumor types was included, encompassing a consistent number of so-called *“cold”* immune-resistant cancers, such as MSS colon-rectal, pancreatic and breast cancer (9, 10, 14). Moreover, the presence of primary brain tumors, always excluded from past series but nowadays more and more present in studies of immunotherapy, enriches and confers elements of novelty to our work (15). Secondly, in our knowledge this is the largest report focusing on patients receiving IMAs-based combinations (n=126/173), that are currently explored in ep-CTs to counteract primary and acquired resistance to PD-(L)1 blockade (9, 10, 14, 16). Finally, the median follow-up of 46.6 months is the longest reported in literature so far, giving higher consistency to our results when compared to other existing scores (9, 10, 14).

Across baseline blood variables, NLR>6 correlated with decreased survival, potentially mirroring higher systemic inflammation and a more aggressive disease, as already described for NSCLC, melanoma, renal cell cancer and GRIm-Score cohort (9, 17–19). Serum albumin, both as continuous variable and punctual value of ≥3.5 g/dL, was associated to improved outcome, reflecting a healthy physical condition with adequate nutritional status and possibly more efficient immune response (20–22). In contrast to most of the previous experiences, LDH above the upper value limit did not seem prognostic in our cohort (9, 10). Indeed, the discriminating power of markers of necrosis and disease burden may be reduced by the inclusion of more immune-resistant and aggressive tumors. The PD-L1 tissue expression is a well-recognized predictive biomarker for immunotherapy with PD-(L)1 inhibitors in a range solid tumors such as NSCLC, HNSCC and gastric cancer (23). We explored its predictive significance in our cohort of patients treated with IMAs-based experimental therapies, without finding any statistically significant difference in survival after stratification for PD-L1 expression. This observation is not surprising and, despite coming from an explorative analysis on a small number of patients, it might reinforce the concept to test this biomarker only in specific tumors, avoiding its use in a heterogeneous, unselected population.

With explorative purpose only, we also performed a survival analysis excluding patients treated with anti-PD-(L)1 and anti-CTLA-4 doublets, already approved in clinical practice and known to improve outcome in several cancer types (11–13). Interestingly, patients treated with combinations of different IMAs seemed to have shorter survival compared to those receiving IMAs single-agent or combined with TKIs or chemotherapy. This analysis is not adequately powered and speculative in nature, but may contribute to question the role of next-generation immunological targets in improving benefit over PD-(L)1 blockade alone, as observed in recent experiences (24, 25). Another hypothesis-generating observation suggested longer survival for patients developing G2-4 ir-AEs during treatment. Although this has been already described for anti-PD-(L)1 agents, it is worth observing a similar pattern in our heterogeneous cohort of patients treated with IMAs-based combinations (26).

In the MVA, pre-treatment serum albumin as continuous variable, line of treatment and number of metastatic sites confirmed their independent prognostic significance. The early administration of IMAs, when the disease burden is low, the tumor biology is less aggressive and the immunological status expressed by serum albumin is preserved, seem to improve clinical outcome (1, 20–22). This reinforces the concept to propose a modern clinical trial as soon as possible during the disease course to maximize the therapeutic intent (1). After splitting albumin value at the median (3.84 g/dL), we built a scoring system which stratified patients in three groups with increasing score associated to longer survival. Of note, this score proved to be reproducible and consistent in an external cohort of patients. Compared to previous experiences, such as the GRIm-Score, our score was derived from a large training cohort including many different tumor types and IMAs-based combinations, better reflecting the new wave of ep-CTs. Interestingly, a score of 0 correlated to very short survival (mOS: 2.3 months), even below the threshold usually required for the inclusion into a clinical study. This suggests that more efficient methods of patients’ selection are strongly needed. Although several experiences focused on biomarkers to predict benefit from IMAs, such as specific T cells subpopulations, these parameters are difficult to obtain and use in daily practice (27, 28). To meet this purpose, we built a handy nomogram based on variables significant in the MVA. Our nomogram is designed as a ready-to-use prognostic tool during patient’s screening, helping physicians in selecting who may gain the maximal benefit from the inclusion into immunotherapeutic ep-CTs. Certainly, the nomogram should not substitute complex clinical judgement, but it could be worth integrating into the clinical decision process.

Our study has several limitations, as it is retrospective in its nature, needing prospective validation. However, confirming the significance of our scoring system in an external cohort of patients with different characteristics, supports its prognostic power for a broad application in clinical practice. Secondly, even if the prognostic significance of clinical variables such as serum albumin, line of treatment and number of metastatic sites could be expected in patients with advanced cancer it is of value establishing their relevance for the first time in a large population of patients treated with modern IMAs-based combinations within ep-CTs. A third limitation is that the discriminating performance of the nomogram obtained appeared moderate (HCI 0.63), nevertheless, the larger sample size and the longer follow-up in comparison to previous experiences confer high reliability to our results (29, 30). Lastly, the inclusion of patients receiving a wide range of different therapies and presenting with rare histologies (such as GBM) could lead to several potential biases. Nevertheless, we suggest that a prognostic nomogram derived from a heterogeneous population, including primary brain tumors as well, strongly reflects modern era of next-generation ep-CTs and remains valuable, offering clinicians a universal tool for patients’ selection in the daily practice of a Phase I Unit.

In summary, we explored the prognostic significance of several variables in patients with advanced cancers treated at our Institution within modern IMAs-based early-phase studies. We developed a scoring system based on three variables (serum albumin, number of metastatic sites and line of treatment), obtaining three groups with different survival. The significance of this score was confirmed in an external cohort. In conclusion, from these factors we built a ready-to-use nomogram for clinical practice. In the modern landscape of immunotherapeutic ep-CTs, this prognostic tool can help to better stratify patients’ prognosis at trial enrollment, maximizing the therapeutic intent.

## Data availability statement

The raw data supporting the conclusions of this article will be made available by the authors, without undue reservation.

## Ethics statement

The studies involving humans were approved by Ethical committee of IRCCS Humanitas Research Hospital. The studies were conducted in accordance with the local legislation and institutional requirements. The human samples used in this study were acquired from a by- product of routine care or industry. Written informed consent for participation was not required from the participants or the participants’ legal guardians/next of kin in accordance with the national legislation and institutional requirements.

## Author contributions

AL: Conceptualization, Data curation, Investigation, Methodology, Writing – original draft, Writing – review & editing. AD: Conceptualization, Data curation, Investigation, Methodology, Writing – original draft, Writing – review & editing. LG: Data curation, Formal analysis, Methodology, Writing – original draft. PP: Data curation, Writing – review & editing. EL: Data curation, Writing – review & editing. ADM: Data curation, Writing – review & editing. CB: Data curation, Writing – review & editing. JK: Data curation, Writing – review & editing. AM: Data curation, Writing – review & editing. OM: Data curation, Writing – review & editing. BS: Data curation, Writing – review & editing. DP: Resources, Supervision, Validation, Writing – review & editing. AS: Resources, Supervision, Validation, Writing – review & editing. MS: Conceptualization, Resources, Supervision, Validation, Writing – review & editing.
