# Supplementary material for: Refining patient selection for next-generation immunotherapeutic early-phase clinical trials with a novel and externally validated prognostic nomogram
Source: Front Immunol. 2024 Jan 15;15:1323151. doi: 10.3389/fimmu.2024.1323151 (PMC10828843; doi:10.3389/fimmu.2024.1323151)
Supplement: Supplementary file 1 [file Image_1.pdf]

S1

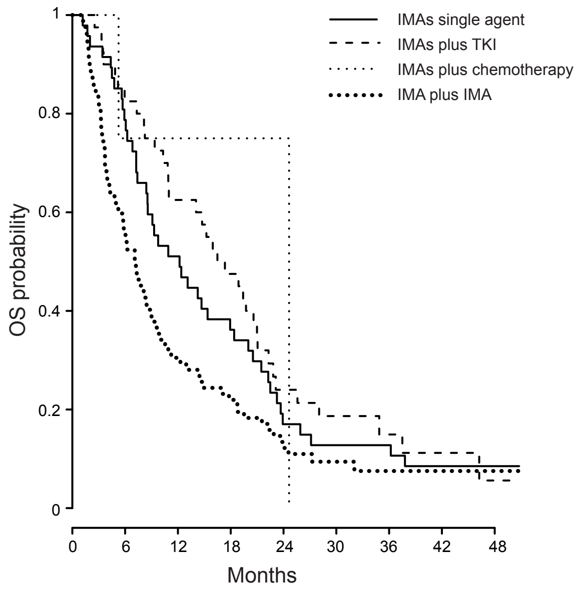

Supplementary Figure 1. OS Kaplan-Meyer curve according to type of treatment.
IMAs: immunomodulating agents; TKI: tyrosine kinase inhibitor
