# Supplementary figures and images for: Refining patient selection for next-generation immunotherapeutic early-phase clinical trials with a novel and externally validated prognostic nomogram
Source: Front Immunol. 2024 Jan 15;15:1323151. doi: 10.3389/fimmu.2024.1323151 (PMC10828843; doi:10.3389/fimmu.2024.1323151)

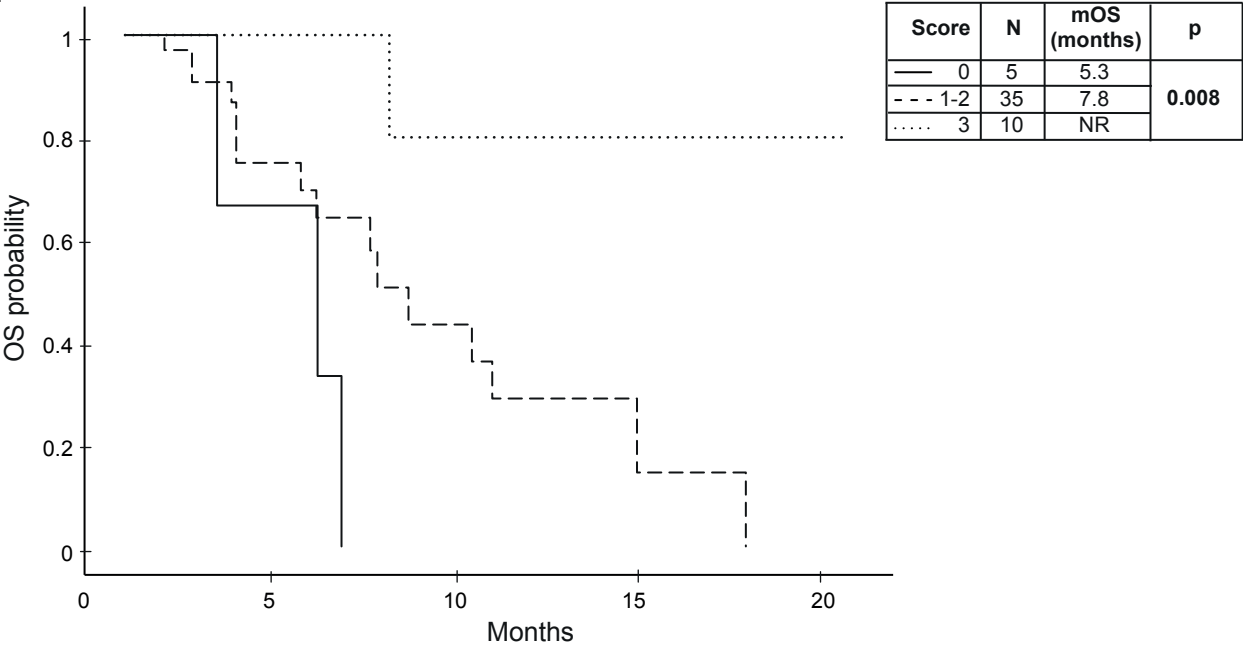

Supplementary Figure 2. OS Kaplan-Meyer curves for the validation cohort.

Supplement: Supplementary file 2 [file Image_2.pdf]
