# Supplementary material for: Refining patient selection for next-generation immunotherapeutic early-phase clinical trials with a novel and externally validated prognostic nomogram
Source: Front Immunol. 2024 Jan 15;15:1323151. doi: 10.3389/fimmu.2024.1323151 (PMC10828843; doi:10.3389/fimmu.2024.1323151)
Supplement: Supplementary file 3 [file Table_1.docx]

| List of variables |
| --- |
| Sex |
| PD-L1 expression |
| Steroid dose |
| Age |
| ECOG PS |
| Type of treatment |
| Line of treatment |
| Number of metastatic sites |
| Metastatic sites |
| Nodes |
| Liver |
| Lung |
| Bone |
| CNS |
| Blood count |
| Hemoglobin |
| Platelets count |
| Total white blood cells count |
| Neutrophils absolute count |
| Lymphocytes absolute count |
| Eosinophils absolute count |
| Monocytes absolute count |
| NLR |
| Serum albumin |
| Serum protein |
| TRAEs |
| ir-AEs G2-4 |
| ir-AEs G3-4 |

**Supplementary Table 1.** **List of variables analyzed.**

ECOG PS: Eastern Cooperative Oncology Group performance status; NLR: neutrophil-to-lymphocyte ratio; TRAEs: treatment-related adverse events; ir-AEs: immune-related adverse events
