# Supplementary material for: Refining patient selection for next-generation immunotherapeutic early-phase clinical trials with a novel and externally validated prognostic nomogram
Source: Front Immunol. 2024 Jan 15;15:1323151. doi: 10.3389/fimmu.2024.1323151 (PMC10828843; doi:10.3389/fimmu.2024.1323151)
Supplement: Supplementary file 4 [file Table_2.docx]

| Target | N |
| --- | --- |
| PD-1 | 27 |
| PD-L1 | 2 |
| CSFR1R | 11 |
| IL1-beta | 5 |
| TGFbeta-R | 2 |
|  |  |
| IL1-beta/chemotherapy | 4 |
|  |  |
| PD-1/c-MET | 7 |
| PD-1/VEGFR-MET-AXL | 24 |
| PD-1/CRBN | 1 |
| PD-1/CTLA-4/VEGFR-MET-AXL* | 5 |
| PD-1/VEGFR | 3 |
|  |  |
| PD-L1/CD38+ | 18 |
| PD-1/CSFR1R | 11 |
| PD-1/IL-8 | 10 |
| PD-1/CTLA-4* | 13 |
| PD-1/IDO1 | 2 |
| PD-1/IL1-beta | 4 |
| PD-1/mesothelin | 2 |
| PD-1/NRP1 | 1 |
| PD-1/TGFbeta | 15 |
| PD-1/TIGIT | 1 |
| PD-1/TIM-3 | 5 |

**Supplementary Table 2. Targets of experimental agents.**

* Removed from exploratory survival analysis
