# Supplementary material for: Refining patient selection for next-generation immunotherapeutic early-phase clinical trials with a novel and externally validated prognostic nomogram
Source: Front Immunol. 2024 Jan 15;15:1323151. doi: 10.3389/fimmu.2024.1323151 (PMC10828843; doi:10.3389/fimmu.2024.1323151)
Supplement: Supplementary file 5 [file Table_3.docx]

| Treatment related adverse events (G2-4) | N |
| --- | --- |
| Transaminitis | 18 |
| Fatigue | 14 |
| Colitis/diarrhea | 14 |
| Rash | 12 |
| Hand-foot syndrome | 11 |
| Hypertension | 9 |
| Nausea | 6 |
| Hyperlipasemia | 5 |
| Hypothyroidism | 5 |
| Hyperthyroidism | 4 |
| Hyperamylasemia | 4 |
| Neutropenia | 3 |
| Stomatitis/Mucositis | 3 |
| Vomiting | 3 |
| Infusion reaction | 3 |
| Proteinuria | 2 |
| Peripheral neuropathy | 2 |
| Arthralgia/Myalgia | 2 |
| CPK increase | 2 |
| Interstitial pneumonitis | 2 |
| Hypophysitis | 1 |
| Myocarditis | 1 |
| Diabetes mellitus | 1 |
| QTcF prolongation | 1 |
| Squamous cell carcinoma | 1 |

**Supplementary Table 3.** **Frequency of main treatment-related adverse events (G2-4).**
