# Supplementary material for: Refining patient selection for next-generation immunotherapeutic early-phase clinical trials with a novel and externally validated prognostic nomogram
Source: Front Immunol. 2024 Jan 15;15:1323151. doi: 10.3389/fimmu.2024.1323151 (PMC10828843; doi:10.3389/fimmu.2024.1323151)
Supplement: Supplementary file 6 [file Table_4.doc]

|  |  | **Serum albumin** |  | **Line of treatment** |  | **N° of metastatic sites** |  |
| --- | --- | --- | --- | --- | --- | --- | --- |
|  | N | median | range | median | range | median | range |
| **All** |  | 3.84 | 2.7-4.8 | 2 | 2-9 | 2 | 1-6 |
| **Score 0** | 9 | 3.4 | 2.7-3.7 | 4 | 4-7 | 3 | 3-5 |
| **Score 1** | 104 | 3.7 | 2.8-4.7 | 3 | 2-9 | 2 | 1-6 |
| **Score 2** | 35 | 4.1 | 3.9-4.8 | 2 | 2-3 | 1 | 1-2 |

**Supplementary Table 4.** **Variables distribution for each prognostic level.**
