# Supplementary material for: Refining patient selection for next-generation immunotherapeutic early-phase clinical trials with a novel and externally validated prognostic nomogram
Source: Front Immunol. 2024 Jan 15;15:1323151. doi: 10.3389/fimmu.2024.1323151 (PMC10828843; doi:10.3389/fimmu.2024.1323151)
Supplement: Supplementary file 7 [file Table_5.docx]

|  |  |
| --- | --- |
| Variable |  |
|  |  |
| Age, median (range) | 61.5 (25.3-89.3) |
|  |  |
|  | **N (%)** |
| Sex |  |
| Male | 21 (42) |
| Female | 29 (58) |
| Tumor type |  |
| Ovarian cancer | 11 (22) |
| Cervical cancer | 9 (18) |
| Head & Neck cancer | 6 (12) |
| HCC | 4 (8) |
| Gastro-esophageal cancer | 4 (8) |
| NSCLC | 3 (6) |
| CRC | 3 (6) |
| Hepatobiliary tract cancer | 2 (4) |
| Others | 8 (16) |
| Therapy type |  |
| Immunotherapy combo | 8 (16) |
| Immunotherapy single agent | 36 (72) |
| Immunotherapy + TKIs | 6 (12) |

**Supplementary Table 5.** **Imperial College** **validation** **cohort main clinicopathological characteristics.**

HCC: hepatocellular carcinoma; NSCLC: non-small cell lung cancer; CRC: colorectal cancer; TKIs: tyrosine kinase inhibitors
